# Oral health of chemotherapy patients before and after provision of oral hygiene instructions at a tertiary care hospital: pre-post design

**DOI:** 10.1186/s12903-024-04093-0

**Published:** 2024-06-04

**Authors:** Mashal Amin, Farhan Raza Khan, Asad Allana, Rubina Barolia, Iqbal Azam

**Affiliations:** Aga Khan University, Stadium Road, Karachi, 74800 Pakistan

**Keywords:** Oral health, Chemotherapy, Oral Hygiene instructions, Dentists

## Abstract

**Objective:**

Disturbances in the oral mucosa is a major concern among patients undergoing chemotherapy. One of the most significant barriers in the implementation of oral care is the lack of knowledge. The aim of the study was to assess gingival and periodontal health status of chemotherapy patients before and after the provision of oral hygiene instructions.

**Methods:**

A single group, pre-post test was conducted to assess oral health status of patients at the daycare chemotherapy, Aga Khan University Hospital, Karachi, Pakistan. Oral hygiene instructions were given with study models and leaflets. Patients were followed for 6-weeks. Oral health was assessed by using Simplified-Oral Hygiene Index (OHI-S) and Community Periodontal Index (CPI). Differences in indices were analyzed in STATA version-15.0 using Generalized Estimating Equation (GEE) and Wilcoxon Signed-rank test.

**Results:**

Out of 74, 53 (72%) patients completed study follow-up. Improvement in the OHI-S was found in 14 (26%) patients (*p*-value < 0.001). GEE showed that age [adjusted OR = 1.10; 95% CI: 1.03–1.11], current chemotherapy cycle [adjusted OR = 1.19; 95% CI: 0.98–1.46], highest education level [Adjusted OR = 1.37; 95% CI: 1.08–12.7] and cancer therapy [Adjusted OR = 0.12; 95% CI: 0.24–0.55] were significantly associated with the change in OHI-S. Wilcoxon signed-rank test showed positive changes in the CPI (*p*-value < 0.001).

**Conclusions:**

Basic oral hygiene instructional intervention can be effective in improving the oral hygiene of chemotherapy patients. Nurses should also play a key role in providing psychological and nutritional support to patients.

**Supplementary Information:**

The online version contains supplementary material available at 10.1186/s12903-024-04093-0.

## Clinical relevance

### Scientific Rationale for the study

This study aims to assess the gingival and periodontal health status among chemotherapy patients to understand the prevalence and severity of oral complications, enabling the development of targeted interventions to improve their oral care and overall quality of life.

### Principal findings

This study demonstrates that while a significant proportion of cancer patients maintained poor oral hygiene, overall, there was a significant improvement in the oral hygiene ratings of all participants.

### Implications

Nurses could endorse patient satisfaction, aid with behavioral and physical interventions, and can encourage patient compliance with therapy and required follow-up.

## Introduction

The first endeavor at employing chemotherapy in oncology was in 1942, when mustard nitrogen was administered to treat malignant lymphoma [1]. Since then, several attempts have been made to enhance chemotherapeutic medicines, assess their actions, and integrate their usage. Nevertheless, the advancement of chemotherapeutic processes and chemicals may result in side effects that impede the patients’ lives as well as the therapy of collateral disorders [2]. A diverse range of bacteria thrive in the oral cavity and oral cavity acts as a harbor to them and there is a possibility of acquiring oral complications during cancer treatment [3]. The degree of impact on normal tissues appears to be proportional to the dose of the antineoplastic medicine employed, as well as the frequency with which the agent is administered [4]. Many medications target quickly proliferating cells; nevertheless, they have a similar impact upon rapidly proliferating normal tissues. They damage the basal cells of the mucosal layer, specifically in the oral mucosa, and their replenishment or turnover is impaired, culminating in mucosal ulceration [5]. It is predicted that more than 30–35% of cancer patients suffer from conditions that affect their overall health and quality of life. Not all cancer chemotherapy patients are equally vulnerable to oral complications. A variety of factors that influence the frequency and severity of oral complications associated with therapy have been established [6]. Oral consequences of cancer chemotherapy involve both initial problems including inflammation and bleeding of gums, reduction in salivary flow, taste disturbances, bacterial and candida infections etc. and delayed problems such as atrophy of the mucosa and dryness of mouth. Patient-related factors encompass a range of variables that can influence the development and severity of oral complications during cancer therapy. These variables include the specific tumor diagnosis, the age and gender of the patient, the oral health condition before initiating cancer therapy, the level of oral care administered during treatment, as well as baseline factors such as pre-existing xerostomia (dry mouth) and neutrophil counts. Notably, individuals diagnosed with hematologic neoplasms, such as leukemia and lymphoma, face a heightened risk of experiencing oral complications compared to those with solid tumors. However, an exception to this trend is observed in patients with head and neck tumors, where the risk of developing oral issues is also notable. Among patients undergoing chemotherapy, disturbances in the function of the gastrointestinal mucosa are a significant concern [7]. Mucositis, a transient adverse effect of chemotherapy, can affect the upper gastrointestinal tract, manifesting in symptoms ranging from dry mouth to the development of oral ulcers and discomfort during swallowing [8]. Substandard oral hygiene practices have been generally related to a greater possibility for oral toxicity [9].

Numerous research studies have explored the advantages of receiving professional oral care to prevent oral mucositis (OM), but only a limited number of them have placed significant emphasis on educating patients in this regard [10, 11]. Therefore, difficulty in the implementation of standard oral hygiene practice persists especially among cancer patients. One of the most significant barriers in the implementation of oral care is the lack of knowledge [12]. The purpose of dissemination of oral hygiene education to the patients is to enlighten them about the fundamental aspects of their oral health and attempt to change their behavior and encourage them to continue improving their own health [13]. Improving oral health education, behavior change and maintaining excellent oral hygiene are the key targets of oral health educational programs [14]. This study’s rationale highlights the potential benefits for both clinical practitioners and patients. By focusing on education and assessing the impact of oral hygiene instructions, it aims to enhance patient outcomes, prevent complications, improve patient compliance, offer personalized care, contribute to better healthcare delivery, reduce costs, and, most importantly, enhance the quality of life for chemotherapy patients undergoing cancer treatment. Therefore, the aim of this study was to assess gingival and periodontal health status among chemotherapy patients before and after the provision of oral hygiene instructions.

## Methodology

### Study design, study settings and eligibility

We conducted a single group pre and post-test design (Quasi-Experimental) study from May 2019 to July 2019 at one of the major tertiary care hospitals with daycare chemotherapy unit in Karachi, Pakistan. All adult cancer patients (18 years and above) undergoing chemotherapy and willing to participate in the study were enrolled. However, patients with head & neck cancer, hematopoietic stem cell transplants, presence of any disease in or around the mouth, patients with any physical disability or diagnosed and currently under treatment for any mental illness (such as depression, dementia, delirium), pregnant or breastfeeding women and patients who had less than 6 weeks to complete their chemotherapy were excluded from the study. Sample size estimation was done using NCSS PASS software version 19.0. A sample of 67 was found to achieve the power of 0.8 at significance level (alpha) of 0.05 to detect mean of paired differences of 0.2 with an estimated standard deviation of differences of 0.3, using a two-sided paired t-test. A non-response rate of 10% was added, and the final sample size to be achieved was 74 patients, using non-probability, purposive sampling technique.

### Intervention, outcome, and ethical considerations

At baseline, dental history was taken, and oral health status of the patients was assessed by trained dental hygienist followed by oral hygiene instructions given via face-to-face counseling using dental study models, oral hygiene information leaflets and a short video made in the native language. Assessment of oral health status was repeated after 6 weeks of intervention using two oral health indices to measure the gingival and periodontal health status. For gingival health assessment, we used “Simplified Oral Hygiene Index” and for the periodontal health assessment, “CPITN” (Community Periodontal Index of Treatment Needs) was employed [15, 16]. Simplified oral hygiene index is a straightforward method for evaluating oral hygiene by assessing soft debris and calculus deposits on the teeth and gums. CPITN, developed by the World Health Organization, is a comprehensive tool for assessing periodontal health and categorizing treatment needs [17]. These assessments allowed for a comprehensive evaluation of the patients’ oral health status, including both cleanliness and periodontal health.

Study was initiated once the permission from the ethical review committee of the Aga Khan University Hospital was obtained. Data was collected after obtaining written informed consent in the native language from the participants. Oral hygiene instructions were modified for patients with Platelet count < 70,000 cells/microL or Absolute neutrophil count < 1000 cells/microL. These tests are routinely done for patients undergoing chemotherapy. Patients with low platelets or neutrophil counts were advised to skip flossing in their routine hygiene care to avoid bleeding from gums.

### Statistical analysis

Data were analyzed with STATA version 15.0. For descriptive analysis, frequencies/proportions were computed to assess the distribution of qualitative variables (gender, education, marital status, comorbidities, primary site of cancer, stage of cancer, frequency of brushing and flossing and last visit to the dentist). Measures of central tendency were computed for the quantitative variables. Due to non-normal distribution, median and Interquartile range was computed for the age, height, weight, total and current cycles of chemotherapy in weeks and frequency of chemotherapy.

For inferential analysis, two groups of patients were compared for all their characteristics using Pearson Chi-square test or Fisher’s exact (where applicable) and Mann-Whitney U test to compare the two medians. The Oral Hygiene Index was analyzed using Generalized Estimating Equation. Bivariate analysis was run, keeping the cutoff *p*-value less than or equal to 0.25. Stepwise model building was done for multivariable analysis and all the plausible interactions were assessed.

## Results

After the initial screening of 106 chemotherapy patients, 76 patients were eligible for our study. Out of the 76 eligible patients, 74 patients were enrolled in the study after taking the written informed consent. The other two patients refused to participate. Of 74 enrolled patients, 53 (72%) completed the study follow-up period. Three patients died during the follow-up period of the study, whereas 18 patients were lost to follow-up, mainly because their chemotherapy ended.

The median age of the enrolled patients was 49 years with interquartile ranges of 19. Of 74 participants 52(71%) were females. The highest level of education achieved was bachelor’s and above for 37(50%) participants. Fifty-one (69%) were unemployed and the predominant ethnicity was muhajir 34(46%). The median of total planned cycles of chemotherapy was 7.5 (IQR = 5) and the median of current chemotherapy cycle was 3 (IQR = 5). Practice of tooth brushing was reported by 64(86.5%) participants, out of which only 28(44%) patients reported practice of tooth brushing twice daily. Flossing was reported to be practiced by only 8(11%) participants. Only 5(7%) patients reported visiting a dentist during the last 6 months. Surprisingly, 18(24%) participants have never visited a dentist in their life. (Table 1)

**Table 1:** Baseline characteristics of the study participants (*n* = 74)

VARIABLES	CATEGORIES or [central tendency)	Count (%)
Age	[Median (IQR)]	49 (19)
Gender	Female Male	52 (70.27%) 22 (29.73%)
Weight in pounds	[Median (IQR)]	160 (12)
Height in inches	[Median (IQR)]	72.4 (16.5)
Marital Status	Married Single	65 (87.8%) 9 (12.2%)
Highest level of education achieved	Bachelors & above Metric or Intermediate Below metric	37 (50%) 10 (13.5%) 27 (36.5%)
Employment status	Employed Unemployed Student	18 (24.3%) 51 (69.0%) 5 (6.76%)
Primary cancer site	Breast Lung Ovary Others	34 (45.9%) 7 (9.46%) 6 (8.11%) 27 (36.5%)
Cancer stage	Stage I Stage II Stage III Stage IV	12 (16.2%) 21 (28.4%) 21 (28.4%) 20 (27.0%)
Cancer therapy	Only chemotherapy Chemo & radiation therapy	69 (93.2%) 5 (6.76%)
Co-morbidities	No Yes	41 (55.4%) 33 (44.6%)
Total cycles of chemo	[Median (IQR)]	7.5 (5)
Current chemo cycle	[Median (IQR)]	3 (5)
Frequency of cycle	[Median (IQR)]	3 (1)
Brushing	Yes No	64 (86.5%) 10 (13.5%)
Frequency of brushing	Once-daily Twice-daily	36 (56.3%) 28 (43.7%)
Flossing	Yes No	8 (10.8%) 66 (89.2%)
Frequency of flossing	Daily More than once a week Once a week	2 (25%) 3 (37%) 3 (35%)
Last dental visit	Less than 6 months More than 6 months More than 1 year Never	5 (6.76%) 7 (9.46%) 44 (59.5%) 18 (24.3%)
Dryness of mouth	Yes No	- 74 (100%)

Out of the 53 participants who completed the follow-up, improvement in the oral hygiene index (from poor to fair oral hygiene) was found in 14 (26%) subjects (*p*-value < 0.001). Among all independent variables, only age was observed to highly significant (P-value < 0.001). Change in oral hygiene index is found among younger patients (median 33.5 years, IQR 14). No change in oral hygiene index is observed in older age groups (median 53.0 years, IQR 17). All other independent variables are not significantly different among the two groups. (Table 2)

**Table 2:** Factors associated with the change in Oral hygiene index (OHI) from poor oral hygiene to fair oral hygiene of patients undergoing chemotherapy

Variables	Categories	No change in OHI (*n* = 39)	Change in OHI (*n* = 14)	*P*-value
**Age** [Median (IQR)]		53.0 (17)	33.5 (14)	< 0.001*
**Gender**	Female Male	27 (75.0%) 12 (70.6%)	9 (25.0%) 5 (29.4%)	0.73
**Weight** [Median (IQR)]		72.5 (12)	73.8 (18)	0.83
**Height** [Median (IQR)]		160 (12)	158 (22)	0.70
**Marital Status**	Married Single	36 (78.3%) 3 (42.9%)	10 (21.7%) 4 (51.1%)	0.07
**Highest education level achieved**	Bachelors & above Metric or Inter Below metric	18 (66.7%) 5 (71.4%) 16 (84.2%)	9 (33.3%) 2 (28.6%) 3 (15.8%)	0.41
**Employment status**	Employed Unemployed Student	9 (69.2%) 27 (77.8%) 3 (64.5%)	4 (30.8%) 8 (22.2%) 2 (35.5%)	0.70
**Primary cancer site**	Breast Lung Ovary Others	20 (76.9%) 3 (50.0%) 2 (66.7%) 14 (77.8%)	6 (23.1%) 3 (50.0%) 1 (33.3%) 4 (22.2%)	0.55
**Cancer stage**	Stage I Stage II Stage III Stage IV	8 (88.9%) 11 (73.3%) 11 (73.3%) 9 (64.3%)	1 (11.1%) 4 (26.7%) 4 (26.7%) 5 (35.7%)	0.70
**Cancer therapy**	Only chemo Chemo & radio	4 (80.0%) 35 (72.9%)	1 (20.0%) 13 (27.1%)	1.00
**Comorbidities**	No Yes	17 (62.9%) 22 (84.6%)	10 (37.0%) 4 (15.4%)	0.11
**Total chemo cycles** [Median (IQR)]		6.0 (7.0)	7.0 (6.0)	0.68
**Current chemo cycle** [Median (IQR)]		3.0 (6.0)	2.0 (3.0)	0.75
**Frequency of cycle** [Median (IQR)]		3.0 (1.0)	3.0 (1.0)	0.76
**Brushing**	Yes No	34 (72.3%) 5 (83.3%)	13 (27.7%) 1 (16.7%)	1.00
**Flossing**	Yes No	6 (85.7%) 33 (73.0%)	1 (14.3%) 13 (27.0%)	0.81
**Last dental visit**	< 6 months > 6 months > 1 year Never	3 (75.0%) 27 (60.0%) 3 (81.8%) 6 (54.6%)	1 (25.0%) 6 (40.0%) 2 (18.2%) 5 (45.5%)	0.22

The generalized estimating equation analysis revealed that age, marital status, employment status, level of education, cancer stage, cancer therapy, co-morbidities, current chemotherapy cycle and last visit to the dentist were statistically significant variables with *p*- value ≤ 0.25. (Table 3)

**Table 3:** Bivariate analysis of binary outcome fair and poor oral hygiene index using Generalized Estimating Equation

Variables	Categories	Odds ratio (95% CI)	*p*- value
**Age**		1.06 (1.03–1.11)	< 0.001*
**Gender**	Female male	1 0.94 (0.33–2.66)	0.90
**Weight**		0.99 (0.95–1.03)	0.65
**Height**		0.99 (0.94–1.03)	0.67
**Marital Status**	Married Single	1 0.27 (0.09–0.78)	0.01*
**Highest education level achieved**	Bachelors & above Metric or Inter Below metric	1 1.29 (0.24–2.56) 0.67 (0.85–2.19)	0.22*
**Employment status**	Employed Unemployed Student	1 1.43 (0.46–4.43) 0.35 (0.08–1.49)	0.10*
**Primary cancer site**	Breast Lung Ovary Others	1 0.69 (0.17–2.81) 1.56 (0.18–9.61) 1.53 (0.49–4.76)	0.74
**Cancer stage**	Stage I Stage II Stage III Stage IV	1 0.27 (0.29–2.42) 0.27 (0.29–2.42) 0.25 (0.03–2.26)	0.22*
**Cancer therapy**	Only chemo Chemo & radio	1 0.41 (0.12–1.59)	0.19*
**Co-morbidities**	No Yes	1 3.54 (1.16–10.8)	0.02*
**Total cycles of chemo**		1.03 (0.91–1.18)	0.60
**Current cycle of chemo**		1.15 (0.96–1.37)	0.12*
**Frequency of cycle**		1.25 (0.79–1.98)	0.34
**Brushing**	Yes No	1 2.76 (0.34–20.6)	0.34
**Flossing**	Yes No	1 0.39 (0.05–3.25)	0.38
**Last dental visit**	< 6 months > 6 months > 1 year Never	1 2.19 (0.34–9.88) 4.24 (0.35–12.8) 1.44 (0.35–5.99)	0.11*

A stepwise approach in the multivariable analysis was done. This included the following independent variables: age, the current cycle of chemotherapy, level of education and cancer therapy. (Table 4)

**Table 4:** Final model after multivariable analysis of a binary outcome fair and poor oral hygiene index using Generalized Estimating Equation

Variables	Odds ratio (95% CI)	Wald Chi-square (*p*-value)
**Age**	1.10 (1.05–1.15)	24.06 (< 0.001)
**Current cycle of chemo**	1.19 (0.98–1.46)	
**Highest education level achieved**		
Bachelors & above Metric or Inter Below metric	1 1.37 (0.31–5.98) 3.69 (1.08–12.7)	
**Cancer therapy**		
Only chemo Chemo & radio	1 0.39 (0.24–0.55)	

**Fig. 1:**
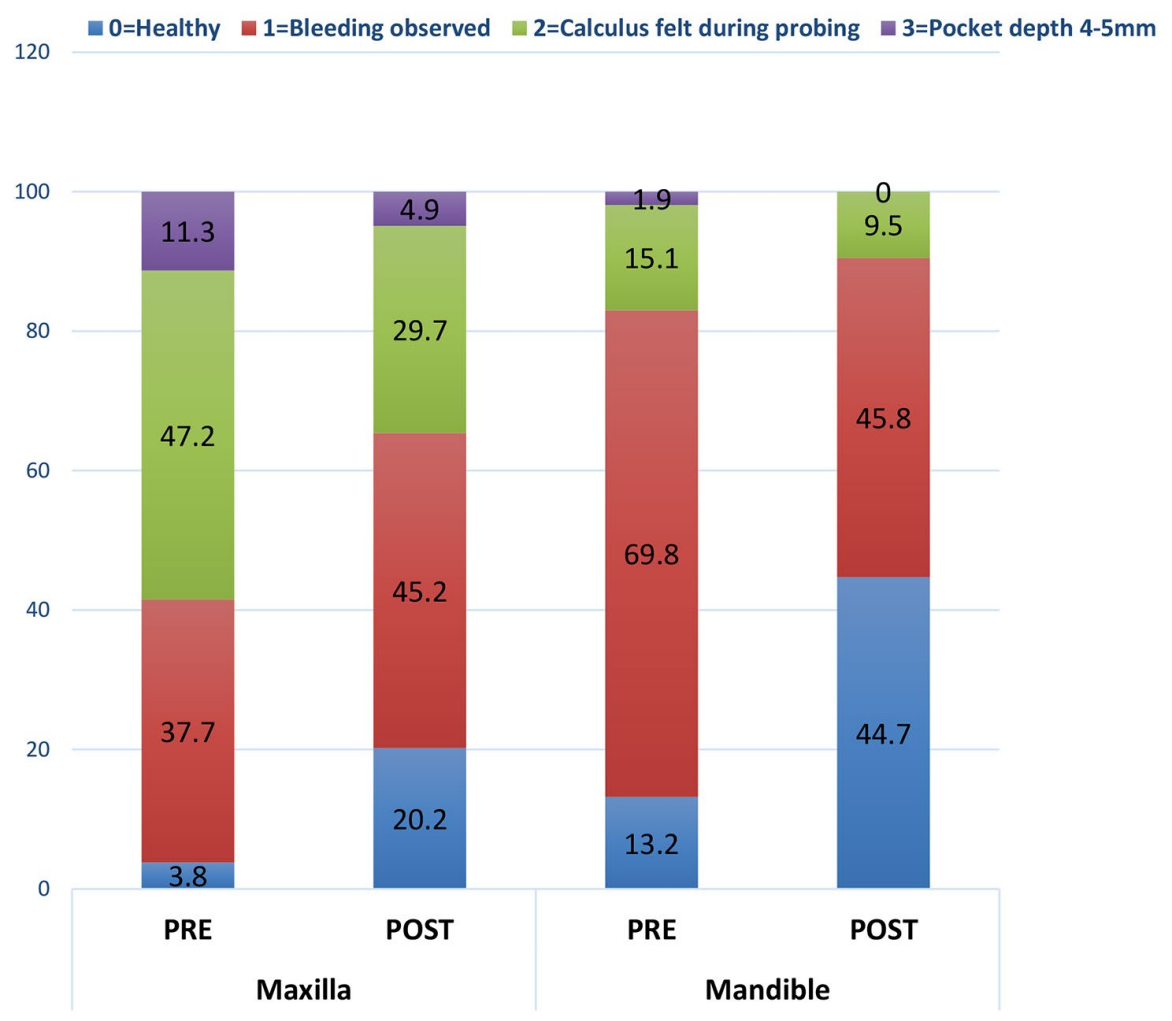
Pre-post changes in maxilla and mandible following oral hygiene instructions

For Community Periodontal Index Treatment Needs (CPTIN), we applied for the Mann-Whitney U test. For maxilla, a remarkable number of patients increased in 0 (healthy) and 1 (mild disease) categories, a moderate decrease in the 2nd (moderate disease) and 3rd (advanced disease; pocket depth 4-5 mm) categories were observed. Whereas for mandible, a remarkable number of patients increased in the healthy category and moderate decrease in the 1st, 2nd and 3rd categories were observed after 6 weeks of oral hygiene instructions. (Fig. 1)

Highly significant Mann-Whitney U test showed that positive changes have been seen in the periodontal index for both maxillary and mandibular teeth. [Maxilla: Z = 8.28 (*p*-value = < 0.001)] [Mandible: Z = 6.96 (*p*-value = < 0.001)]

## Discussion

In the study, we observed an improvement in the oral hygiene index from poor to fair among 26% of the cancer patients whereas 74% remained in the same category of poor oral hygiene. However, there was substantial improvement in the oral hygiene scores of all the cancer patients.

We observed that younger age patients had a low odd of developing poor oral hygiene compared to the older cancer patients. Age was observed as a highly significant variable affecting oral hygiene. Cancer patients who were single demonstrated a low risk of developing poor oral hygiene compared to married subjects, mainly because of the age or lifestyle factors. A study conducted by Guiglia et al. stated that teeth, oral mucosae, alveolar bone, and salivary glands may all experience significant alterations as they age. In elderly people, dental, periodontal, oral mucosal, and salivary illnesses all have a negative impact on their oral health [18].

For patients who are simultaneously being treated with chemotherapy and radiotherapy are actually found to have 88% less risk of developing poor oral hygiene as compared to patients who are only being treated with chemotherapy. This may be because the patients who are also getting treated with radiotherapy are usually receiving a low dose of chemotherapy as compared to patients only being treated with chemotherapy. A study conducted by MUR Naidu et al. also stated that when compared to extended or recurrent administration of lesser dosages of cytotoxic drugs, there is an increased risk of mucositis developing with bolus and continuous infusions [19]. This may be a possible explanation for the protective association of both cancer treatments with poor oral hygiene.

The oral hygiene instruction turned out to be an effective intervention for improvement in oral hygiene among cancer patients. With six weeks of educational intervention, change in the CPITN index was highly significant, which means that the periodontal index in the studied subjects moved from the higher categories (periodontal pockets) to lower categories (towards healthy periodontium). This observation might under or overestimate the effect the educational instruction depending upon baseline oral hygiene of the studied sample. An experimental study conducted by Rodrigo Mariño et al. showed similar results. The study showed that participants who were given interventions like oral health education and oral hygiene instructions outperformed those in the control groups in terms of achievement, experimental groups were considerably more likely than control groups to have better oral health attitudes, oral health knowledge, self-assessed physical health status, self-reported oral hygiene practices, and utilization of oral health services at post-test [20]. Further, the heightened risk of acute oral problems as a result of chemotherapy emphasizes the significance of maintaining adequate oral hygiene habits.

Educating a cancer patient about the significance of oral health is very crucial. But once the patient comprehends the significance of oral health then they certainly adhere to a standard protocol of oral hygiene care. The collaboration of the oncology team with the dental team is imperative for a patient’s health and wellbeing. It is vital that dentists and other oral healthcare providers retain interaction with the oncologist during the length of cancer therapy and seek adequate consultation with the oncologist prior to any dental operations, including prophylaxis [21]. Oral hygiene care has to be reinforced to the patients repeatedly. Educational materials for patients must be available to them for their understanding and knowledge. Including family members in the educational endeavor is equally important because family plays a key role in the cancer patient’s care [22].

Nurses working in oncology settings also have a critical role in assisting the patients in managing their impaired oral function. Nursing care should be designed to promote patient comfort, provide information about pain control to patients and their families, provide information about and assistance with behavioral and physical interventions, prevent and alleviate pharmacologic therapy side effects, and encourage patient compliance with therapy and required follow-up. The nurse should explain why interventions are necessary and provide time for inquiries from the patient and family [23]. Efforts should be made to assess new interventions improving the quality of life in cancer patients. It is imperative to design contemporary educational/instructional models and implement them for the oncology team as well as patients and their caregivers [24].

To the best of our knowledge, this is the first pre-post study conducted to assess oral health status of patients undergoing chemotherapy, involving provision of oral hygiene instructions. We have adapted validated tools for the assessment of study outcomes. The measurement of periodontal parameters was also objective, thus improving the credibility of the findings. However, a major limitation of this study was that it’s a single center study. We did not have a control group to compare the results of the educational intervention. Ethically it was unacceptable to deny essential oral hygiene education to the cancer patients, they needed such intervention the most. Other limitations of this study include: a short follow-up time period (6 weeks is the minimum time period to observe and expect any gingival and periodontal changes). Instructional intervention regarding oral hygiene was only given at the baseline and not during the follow-up time period thus visual reinforcement of the oral hygiene care message was not there. Adherence to oral hygiene practices was not measured in this study. And lastly, we could not report the risk ratios because it was a known limitation of the generalized estimating equation analysis; it reports odds ratios instead. The generalizability of the study is limited, we cannot extrapolate our findings to head & neck cancer patients we had excluded them.

## Conclusion

The study focused on the oral hygiene status of chemotherapy patients found a significant proportion (26%) demonstrated an improvement in oral hygiene index from poor to fair after a six-week oral hygiene instructional intervention. Younger age was strongly associated with positive oral hygiene outcomes, and marital status and cancer therapy were identified as significant factors affecting oral health. In recent times, cancer therapeutic innovations and advancements have significantly enhanced survivability in recent years. As a result, there is an increasing need for continued care of the oral health needs of this group. Basic oral hygiene instructional intervention can be effective in improving the oral hygiene of chemotherapy patients. Our recommendation is to plan and implement large clinical trials on oral hygiene educational interventions for patients undergoing chemotherapy. For early eradication, patients with OM should be given mouth washes, gels, and analgesics. Nurses should also play a key role in providing psychological support and ensuring the patient’s nutritional needs are met. Further, with breakthroughs in computer modelling and deep Learning methods, persons at risk for developing medication toxicities may be recognized and physicians may be better qualified to forecast which patients and treatments are most likely to generate oral adverse effects. Lastly, the effective care of this multifaceted patient group demands multidisciplinary collaborative efforts and the deployment of a holistic, patient-centered strategy with a focus on oral health.

### Electronic supplementary material

Below is the link to the electronic supplementary material.

**Supplementary Material 1: Supplementary Figure.** leaflet for oral hygiene instructions

## Data Availability

The datasets used and/or analyzed during the current study available from the corresponding author on reasonable request.
