# Supplementary figures and images for: Oral health of chemotherapy patients before and after provision of oral hygiene instructions at a tertiary care hospital: pre-post design
Source: BMC Oral Health. 2024 Jun 4;24:655. doi: 10.1186/s12903-024-04093-0 (PMC11149359; doi:10.1186/s12903-024-04093-0)

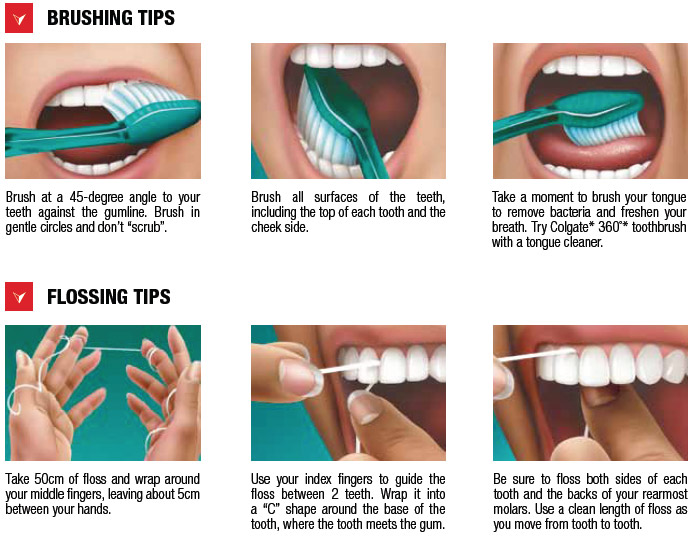


Figure: leaflet for oral hygiene instructions

Supplement: Supplementary file 1 — Supplementary Material 1: Supplementary Figure. leaflet for oral hygiene instructions [file 12903_2024_4093_MOESM1_ESM.docx]
